# Combined strategies for optimal detection of the contact point in AFM force-indentation curves obtained on thin samples and adherent cells

**DOI:** 10.1038/srep21267

**Published:** 2016-02-19

**Authors:** Núria Gavara

**Affiliations:** 1 School of Engineering and Materials Science, Queen Mary University of London, Mile End Road, E1 3NS, London, UK

## Abstract

Atomic Force Microscopy (AFM) is a widely used tool to study cell mechanics. Current AFM setups perform high-throughput probing of living cells, generating large amounts of force-indentations curves that are subsequently analysed using a contact-mechanics model. Here we present several algorithms to detect the contact point in force-indentation curves, a crucial step to achieve fully-automated analysis of AFM-generated data. We quantify and rank the performance of our algorithms by analysing a thousand force-indentation curves obtained on thin soft homogeneous hydrogels, which mimic the stiffness and topographical profile of adherent cells. We take advantage of the fact that all the proposed algorithms are based on sequential search strategies, and show that a combination of them yields the most accurate and unbiased results. Finally, we also observe improved performance when force-indentation curves obtained on adherent cells are analysed using our combined strategy, as compared to the classical algorithm used in the majority of previous cell mechanics studies.

The study of cell mechanics is of fundamental biomedical relevance. In the last decade, a plethora of studies have reported that abnormal cell elasticity is implicated in many human pathologies such as cancer, arthritis, malaria, cardiomyopathies, Alzheimer’s or vascular disease^1^. Atomic Force Microscopy (AFM), is one of the most versatile nanotools to study cell mechanics, because it allows high-resolution mapping of both topography and elasticity of living cells in physiological conditions. Furthermore, thanks to recent novel modelling and measurement protocols, AFM has emerged as a truly quantitative technique to measure cell elasticity^2^,^3^,^4^,^5^. Nevertheless, to become a widespread diagnostic and research tool for biomedicine, measurements of cellular elasticity using AFM need to be high-throughput, accurate and not prone to analysis pitfalls.

Biology-oriented commercial AFM systems include operating modes that perform high-throughput probing of cellular mechanics, by routinely carrying out hundreds of indentations on an area of interest in few minutes. Furthermore, new AFM modes such as force-based imaging combine mechanical mapping and topography imaging performed at the same time^6^. Said approaches can even be combined with simultaneous fluorescence imaging of adherent cells, to provide a correlation between cytoskeletal organization and cell stiffness^7^. Cellular elasticity, formally measured as Young’s modulus (*E*), is obtained by fitting the relationship between cantilever-applied force and resulting sample indentation with a contact-mechanics model that may take into account parameters such as tip shape and sample thickness. There is a major shortcoming in this analytical approach, in that AFM measures only the relative displacement and deflection of the cantilever, but cannot directly measure the gap between the tip and the sample (in non-contact conditions) or the amount of sample indentation (in contact conditions). To obtain said values, as well as an absolute value of cantilever deflection and sample thickness, one needs to identify the point at which the AFM probe comes into contact with the sample, known as the ‘contact point’ (CP). This parameter is not obtained directly in AFM measurements, but rather established through careful inspection of the force-displacement curves output by the AFM. For non-deformable hard surfaces, identification of the contact point is straightforward. But for soft indentable materials, contact point determination is hindered by the poor signal-to-noise ratio of the deflection signal at the initial stages of sample indentation and by the non-linear relationship between cantilever displacement and deflection observed once contact takes place. Inaccurate identification of the contact point leads to erroneous estimates of cantilever force, sample indentation and sample thickness, thus having a paramount effect on the final determination of *E*. Accordingly, previous studies have shown that misidentifying the location of the contact point by 50 nm may lead to estimates of the values of *E* that are incorrect by an order of magnitude^8^.

In recent years, several studies began using automated approaches to identify the contact point of force-displacement (f-d) curves^9^. Those studies have aimed at bypassing subjective user input when estimating the contact point by visual inspection. In addition to improving accuracy, automated approaches are also necessary to handle the large amount of indentation curves generated in current cell mechanics measurements. Several strategies have been proposed to automate the determination of the CP^9^,^10^,^11^,^12^,^13^. Nevertheless, for a given set of indentation data, the optimal strategy to determine the CP will largely depend on experimental conditions, and on factors such as the model used to analyse the data, the shape of the probe, the (non)-adhesive mechanical interaction between the probe and the sample and the (non)-Hertzian behaviour of the sample^10^,^11^. Few studies have compared different strategies to identify the CP^13^,^14^ and all of them have been limited to confirming that different strategies yield comparable results.

This paper focuses on establishing an optimal approach to identify the CP of indentation data obtained in linear elastic, non-adhesive soft thin samples analysed using the BECC model^3^, a correction to the widely used Sneddon’s model for an indenting conical probe^15^. Here we test 4 independent strategies to find the CP, and explore the possibility of combining them to yield superior results. We establish which is the best strategy (and combination of them) by ranking the proposed strategies according to their effectiveness to analyse indentation data obtained on thin soft homogeneous hydrogels, which mimic the stiffness and topographical profile of adherent cells. It should be noted that our findings and recommendations are also applicable to researchers using Sneddon’s model to analyse indentation data obtained on thick samples. Finally, the proposed strategies to identify the CP and our assessment scheme may be useful to researchers facing other experimental conditions and using other contact mechanics models, helping them optimize their own automated CP detection method.

## Experimental Methods

### Preparation of thin polyacrylamide gels

Polyacrylamide gels of graded thickness were prepared as previously described^3^. Initial solutions of 40% acrylamide (A) and 2% bis-acrylamide (B) were diluted in water containing 1 mg/ml of Irgacure 2959, to provide the following concentrations: 5% A and 0.1% B; 8% A and 0.1% B; and 8% A and 0.25% B. A drop of the mix was placed on an activated coverslip and left uncovered, and gel polymerization was started immediately afterwards by exposure to UV light for 10 min.

### Preparation and mechanical characterization of bulk polyacrylamide gels

Bulk polyacrylamide gels were prepared following the concentrations listed above. 3 ml of the polyacrylamide mix were poured on a 35 mm diameter petri dish and gel polymerization was started by exposure to UV light. Petri dishes were left uncovered and exposed to air, in order to mimic the same polymerization conditions carried out for thin samples. Mechanical characterization of bulk gels was performed using a mechanical test system (MTS Bionix 100, MTS, Cirencester, UK) equipped with a 2 mm diameter flat impermeable indenter and a 50 N load cell. The gel was subjected to a compressive indentation rate of 0.01 mm/s, up to a maximum indentation of 10%.

### Cell culture

Cell measurements were performed in living fibroblasts, cell line NIH-3T3 (CCL-1658, ATCC). The culture medium consisted of HEPES-buffered DMEM (Gibco) with 10% calf serum (SAFC Biosciences) and 1:100 Penicillin-Streptomycin (Sigma). For measurements on adherent cells, the sample heater stage was used and set to 32 °C, as higher temperatures gave rise to undesired media evaporation. As previously reported by others^16^, under these culture conditions NIH3T3 display no marked differences in Young’s modulus when proved at 31 °C or 37 °C.

### Measurements

Measurements were performed using a Catalyst AFM (Bruker Corp.) mounted on the stage of an Axiovert 200 inverted microscope (Zeiss) placed on a vibration isolation table (Isostation). V-shaped gold-coated silicon nitride cantilevers with a four sided pyramidal tip (MLCT, Bruker Corp.) were used as probes. The opening angle of the pyramidal tips was assumed to be 35°. The nominal spring constant of the cantilevers was 0.03 Nm^−1^, but each cantilever was calibrated using the thermal fluctuations method^17^ prior to measurements. Measurements were performed on either ultrapure water (for gels) or cell culture medium (for cells) as bathing solution.

Measurements were initiated by calibrating the relationship between photodiode signal and cantilever deflection on a bare region of the glass slide. To automate the acquisition of indentation curves, we used the ‘point-and-shoot’ feature in contact mode to perform line scans along the gel or cell surfaces. Line scans always started over a bare glass location close to the gel/cell edge, so that the recorded initial piezo position at that location could be later used as a zero-height reference to compute sample thickness. Force-displacement curves were obtained at 1 Hz, using 5-μm ramps. When performing measurements on thick gels, the same protocol was used but no initial measurements were performed on bare glass. Gel thickness on those areas was estimated by focusing from the glass surface to the top of the gel surface with the optical microscope, which was equipped with a motorized z-focus.

### Modelling

Force-displacement (*d*-*Z*) curves obtained on thin gels or cells were analysed using the Bottom Effect Cone Correction (BECC)^3^:


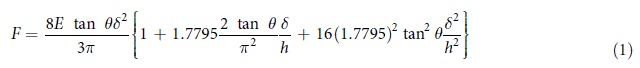


in which *F* is the applied force, δ is indentation, θ is the half-opening angle of the cone, *E* is the Young’s modulus, *h* is the point thickness at that particular location on the sample and the Poisson’s ratio is assumed to be 0.5. As previously discussed^3^, while the BECC could be further extended to model a regular pyramidal tip indentor, approximating a conical indentor doesn’t lead to major differences in the force-indentation relation obtained via the Betti-Rayleigh reciprocal theorem. For thick gels, Sneddon’s model for an indenting cone was used^18^:


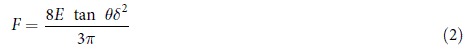


### Data processing

Data processing was carried out using a custom-build analysis code implemented in Matlab (The Mathworks). The code is available upon request. For each force-displacement curve, the contact point was identified using one of the strategies described below. Recorded piezo positions (*Z*) were transformed into sample indentations as:


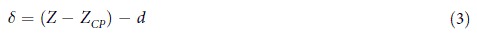


where Z is the displacement of the piezo and Z_CP_ represents the piezo position at the contact point. Sample height was computed as:


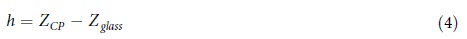


where Z_*glass*_ represents the piezo position at the contact point over a bare region of glass.

It should be noted that only certain regions of the force-displacement curve were used to identify the contact point (depending on the strategy). On the contrary, once CP was identified, the whole δ–F curve was used to compute *E* using nonlinear least-squared fits. Conversely, for measurements performed on adherent cells, only regions of the δ–F curve containing indentations smaller than 600 nm were used for *E* computation, to avoid non-linear elasticity effects. *E* values obtained from all analysed force-displacement curves were used for our study.

## Description of Automated Strategies to Identify CP

The four automated strategies presented here are based on the classical successive search procedure, in which every point (or those within an interval) of the indentation curve is assessed as a trial contact point^4^,^7^,^8^,^9^,^10^,^11^,^13^. For each trial point, a certain ‘test parameter’ is computed, and CP is established as the trial point that yields the maximum (or minimum) value within the tested interval. Each of the four strategies described below is based on using a different test parameter to identify CP. Each test parameter has been chosen because it changes smoothly as the successive search is performed and displays a peak (or valley) in the vicinity of the CP. Importantly, all strategies are aimed at identifying the CP in the approach direction of the indentation curve. In all the following descriptions, the *i* index will indicate the trial points assessed in the successive search procedure, and *i* = 1 will correspond to the data point where the cantilever is furthest away from the sample’s surface.

### Goodness of fit (GoF)

**Figure 1:**
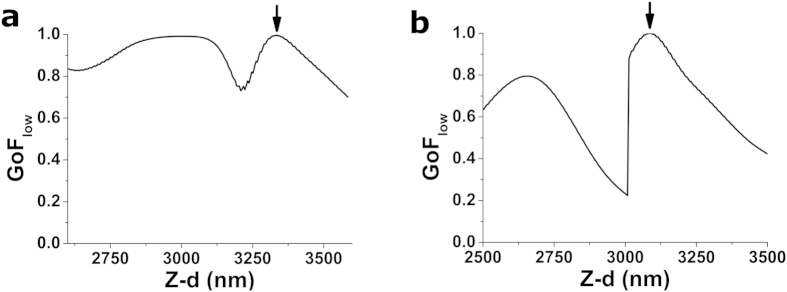
Performance of GoF strategy on two example force-indentation curves (a) and (b), (depicted in Fig.6a and 6b, respectively). Values of GoF for the successive search procedure performed on 100 nm intervals on the vicinity of the contact point. Both force-indentation curves analysed here were obtained on a thin ~10 kPa gel. Strategy depicted is GoF_low_, where only points corresponding to small indentations were used to calculate the fit. Positions of the CP yielded by this strategy are indicated by the black arrows. Plotted GoF values have been normalized using the maximum value obtained within the tested interval.

This is the most standard strategy when using successive search procedures and has been used in the majority of cell mechanics studies reported so far. For each trial point, Z-d data are first converted to δ–F, assuming that δ_i_ = 0 and F_i_ = 0 at the location of the trial point *i*. Then, the appropriate contact-mechanics model is fitted to the contact part of the δ–F data, and either the mean-squared error or the *r*^2^ value of the fit are used as the test parameter. CP is then established as the trial point which yields the lowest value for the mean-squared error^7^, or the highest value for *r*^2 ^13 (Fig. 1). Hermanowicz *et al.* report that when using fits to the whole δ–F curve, computed *E* values are overestimated and their distribution is skewed^4^. This effect is markedly reduced when only the initial part of the δ–F curve is used for *E* computation. Therefore, using only δ–F data points corresponding to low forces (small indentations) is recommended^4^. To assess these claims and compare the performance of both sub-strategies, we have identified the CP in two different ways when using GoF approach. On the one hand, we have used fits to the whole contact region of the δ–F curve (strategy GoF_whole_), and on the other hand, we have fitted only one third of the curve, using the data points corresponding to low indentations (strategy GoF_low_). In both situations, *r*^2^ is used as test parameter (Fig. 1).

### Ratio of Variances (RoV)

On the non-contact part of the indentation curve, there is no mechanical interaction between the probe and the sample. As the cantilever moves towards the sample, no changes in the cantilever deflection are measured, apart from those arising from instrumentation noise. If we were to measure the variance of the deflection signal in a small interval on the non-contact region of the curve, it will be small. Once the tip of the cantilever proceeds to indent the sample’s surface, the increasing force applied by the probe onto the sample’s surface is recorded as a slowly increasing deflection of the cantilever. Therefore, the variance of the deflection signal computed on a small interval within the contact region of the curve will we much larger than that computed in the non-contact region. Importantly, the variances measured in two different intervals belonging both to the non-contact region will be equal, and likewise, the variances measured in two different intervals of the contact region will be similar.

To take advantage of these phenomena, the test parameter RoV is defined as the ratio of the variances of the deflection signal, computed in two small windows to each side of the trial point as:


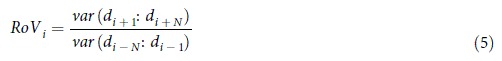


**Figure 2:**
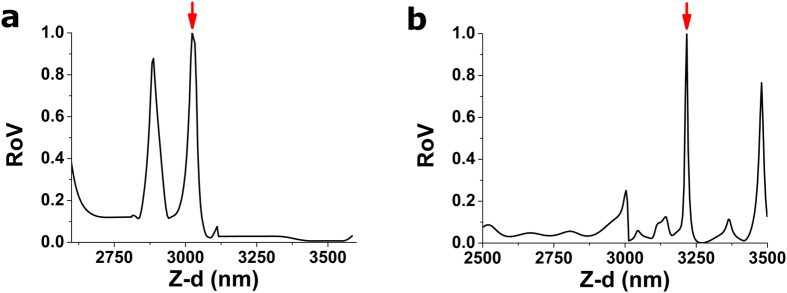
Performance of RoV strategy on two example force-indentation curves (a) and (b), (depicted in Fig. 6a and 6b, respectively). Values of RoV obtained from the successive search procedure, performed on 100 nm intervals on the vicinity of the contact point. Variances to each side of the tested CP were computed using 50 nm windows. Both force-indentation curves analysed here were obtained on a thin ~10 kPa gel. Positions of the CP yielded by this strategy are indicated by the red arrows. Plotted RoV values have been normalized using the maximum value obtained within the tested interval.

For trial points well-within the non-contact region of the curve, RoV will be close to 1 whereas for trial points well-within the contact region, RoV will be slightly larger than 1. Notably, RoV will display a peak in the vicinity of the CP (Fig. 2), which will be readily detected using the successive search procedure.

### Changes in estimated Young’s modulus (ΔE)

**Figure 3:**
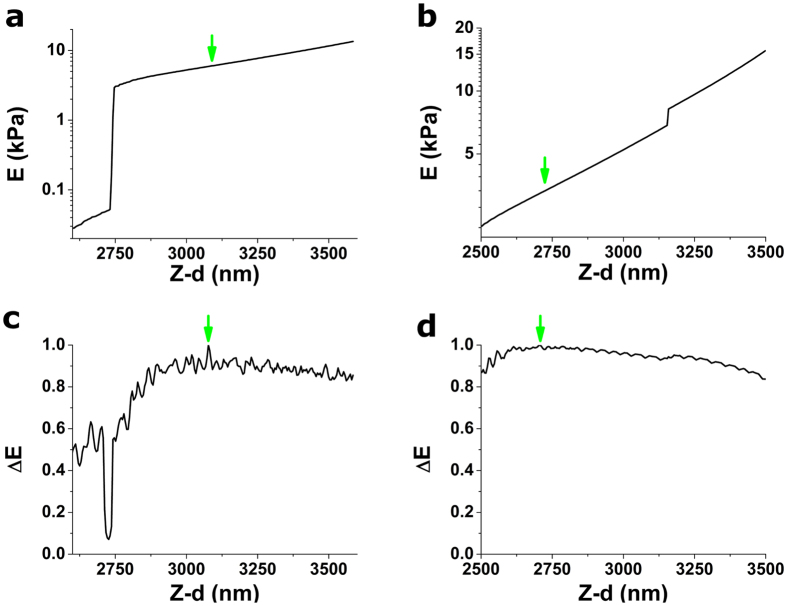
Performance of ΔE strategy on two example force-indentation curves (depicted in Fig. 6). (**a,b**) Young’s modulus obtained when each of the (Z-d) positions on a 100 nm interval are used as candidate contact point. (**c,d**) ΔE test parameter computed using the Young’s modulus values depicted above. d(lnE*)/*d*i* has been computed numerically using central finite differences with a six-order accuracy. Both force-indentation curves analysed here were obtained on a thin ~10 kPa gel. Positions of the CP yielded by this strategy are indicated by the green arrows. Plotted ΔE values have been normalized using the maximum value obtained within the tested interval.

Estimated *E* values depend largely on the final choice of CP. In particular, *E* decreases when parts of the non-contact region are incorporated into the fit, and similarly, *E* increases when the assessed trial point is well-within the contact part region. As previously illustrated^9^, estimated *E* values change monotonically as successive trial points are assessed in sequential search schemes. While d*E/*d*i* appears to be constant when Sneddon’s model is used^9^, an exponential behaviour for d*E/*d*i* is observed when BECC is used to analyse indentation data obtained on thin samples. Upon systematic inspection of *E*(*i*) behaviour on our force-indentation curves, we serendipitously observed a particular feature in the form of a non-stationary inflection point in the vicinity of the CP, which was only manifest when *E*(*i*) was plotted on a logarithmic scale (Fig. 3a,b). To take advantage of this situation and to obtain a test parameter displaying a peak in the vicinity of the CP, we define ΔE as:


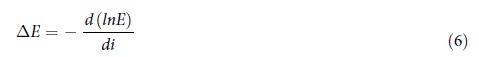


where the minus sign is used solely to turn the inflexion point (a local minima of the d(lnE*)/*d*i* function) into a local maxima, thus giving rise to a peak of the test parameter (Fig. 3c,d).

### Power law exponent (PLE)

**Figure 4:**
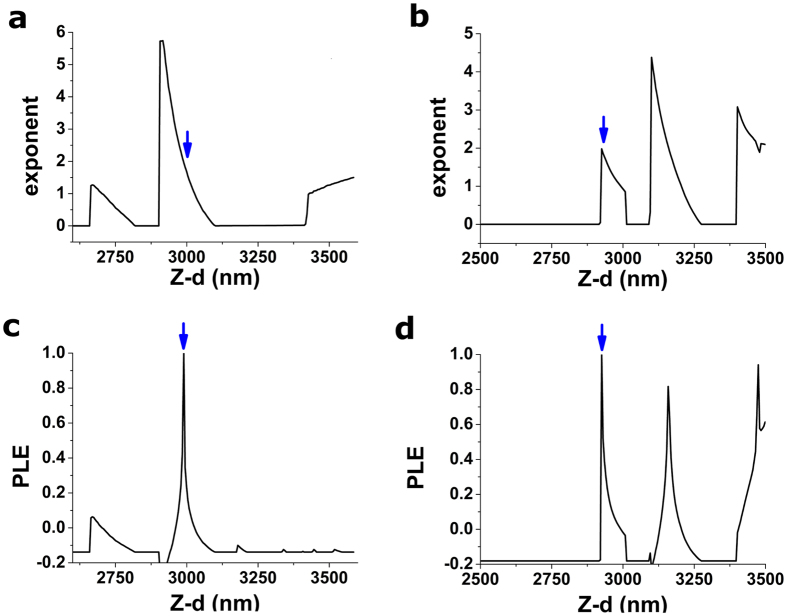
Performance of PLE strategy on two example force-indentation curves (depicted in Fig. 6). (**a,b**) Exponent of the power-law fit obtained when each of the Z-d positions on a 100 nm interval are used as candidate contact point. (**c,d**) PLE test parameter computed using the exponents depicted in a,b. Both force-indentation curves analysed here were obtained on a thin ~10 kPa gel. Positions of the CP yielded by this strategy are indicated by the blue arrows. Plotted PLE values have been normalized using the maximum value obtained within the tested interval.

The relationship between force and sample indentation for a conical indenter is predicted to follow a pure quadratic function^15^ such as 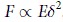. Accordingly, the standard approach described for GoF above fits a quadratic (or polynomial) function to δ–F data, with *E* as the only free parameter. A power law function, such as 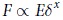 can also be used to fit the indentation data. Such fit will then have *E* and *x* as free parameters. When trial points in the vicinity of the true CP are assessed, the relationship δ–F will follow a pure quadratic function, and thus the fit will yield *x* values close to 2. Nevertheless, for trial points far from the true CP, δ–F will no longer follow a pure quadratic function, and thus *x* values different from 2 will be obtained (Fig. 4a,b). To turn this phenomenon into a function displaying a peak in the vicinity of the CP (Fig. 4c,d), the test parameter PLE is defined as:


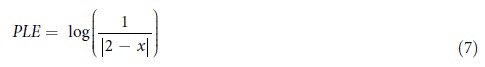


It should be noted that BECC is a correction to Sneddon’s model, and therefore, the pure quadratic relationship between δ–F is predicted only for very small indentations. Similarly, for larger indentations, the higher-order terms arising from the BECC correction will become dominant. Therefore, optimal use of PLE will be hindered if data points corresponding to large indentations are incorporated in the search of the CP. Accordingly, in our algorithm to search for the CP, only a small region (250 nm) in the vicinity of the trial point is fitted, rather than the whole indentation curve. Such precaution will be unnecessary when analysing force-indentation curves obtained on a thick sample using Sneddon’s model.

### Combined strategies

**Figure 5:**
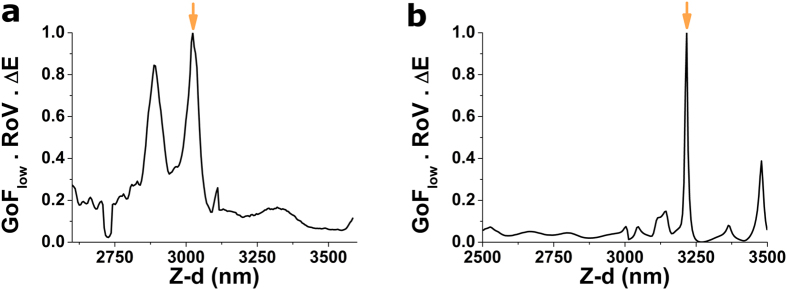
Performance of the best combined strategy on two example force-indentation curves (a) and (b), (depicted in Fig.6a and 6b, respectively). Result of the successive search procedure when strategies GoF_low_, RoV and ΔE are combined, computed as the product of their three test parameters. Both force-indentation curves analysed here were obtained on a thin ~10 kPa gel. Positions of the CP yielded by this strategy are indicated by the orange arrows. Plotted values have been normalized using the maximum value obtained within the tested interval.

All strategies presented above are based on the successive search procedure, and they are designed to display a peak in the vicinity of the contact point. Therefore, it is feasible to combine several of those strategies by using the product of their test parameters as final value to maximize (Fig. 5). Combined strategies may yield more than one peak, thus the CP is established as the one with the highest value.

## Assessment of the Proposed Strategies

**Figure 6:**
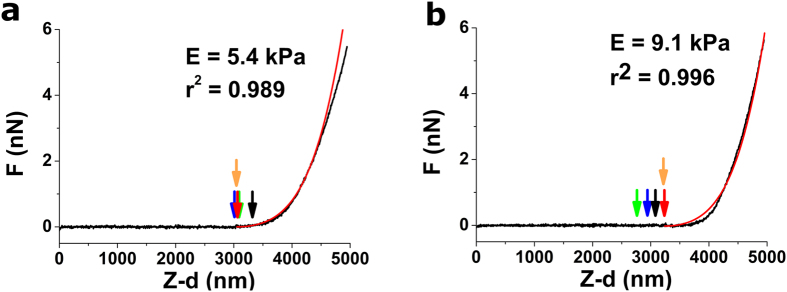
Represenative force-indentation curves with the best fits using BECC model. For both (**a**) and (**b**), black lines are the real data, while red curves represent fits when using the CP obtained by the GoF_low_ ∙ ∆E ∙ RoV strategy. Locations of best CP obtained using each strategy are indicated by arrows (black for GoF, red for RoV, green for ΔE, blue for PLE and orange for GoF_low_ ∙ ∆E ∙ RoV) Both curves were obtained on a thin ~10 kPa gel. Force-indentation curves depicted here have been used to illustrate the performance of the different strategies in Figs 1, 2, 3, 4, 5.

To summarize the key features of an optimal strategy to identify the CP, said strategy must (1) produce results that are accurate, (2) yield a minimal number of outlier values, and also (3) not be impacted by experimental artifacts. In addition, in the case of hydrogels of constant stiffness, *E* values typically follow a normal distribution. Therefore, an extra feature of our results should be that (4) the distribution of *E* values is not skewed. These features can be replaced by quantifiable metrics, in order to rank the performance of the strategies presented above. After analysing a large number of indentations curves carried out on thin samples of constant stiffness (Fig. 6), the distributions of *E* values obtained using each strategy have been measured using the following metrics, in relation to the key features stated above:

(1) Variance, σ^2^(*E*).

(2) Success rate (SR), defined as the number of indentation curves whose analysis yields an *E* value encompassed within a pre-defined range.

(3) Covariance between *E* and point thickness, σ(*E,h*). This is particularly important when measuring thin samples, in which the effect of the stiffer substrate has to be corrected using the BECC model. Once the model is applied, and only if the CP is correctly established, a thin homogeneous sample will display no dependence between point thickness and measured *E*, therefore yielding low covariance between them.

(4) Skewness, *s*(*E*).

Finally, these four parameters are combined into a global metric (*M*), defined as:


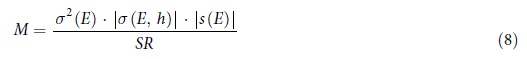


*M* is constructed so that the numerator contains the product of the metrics that a good strategy should minimize, while the denominator contains SR, which is the only defined metric that should be maximized. Accordingly, lower values of *M* will indicate better performance of a strategy.

## Results

### Performance of strategies

**Figure 7:**
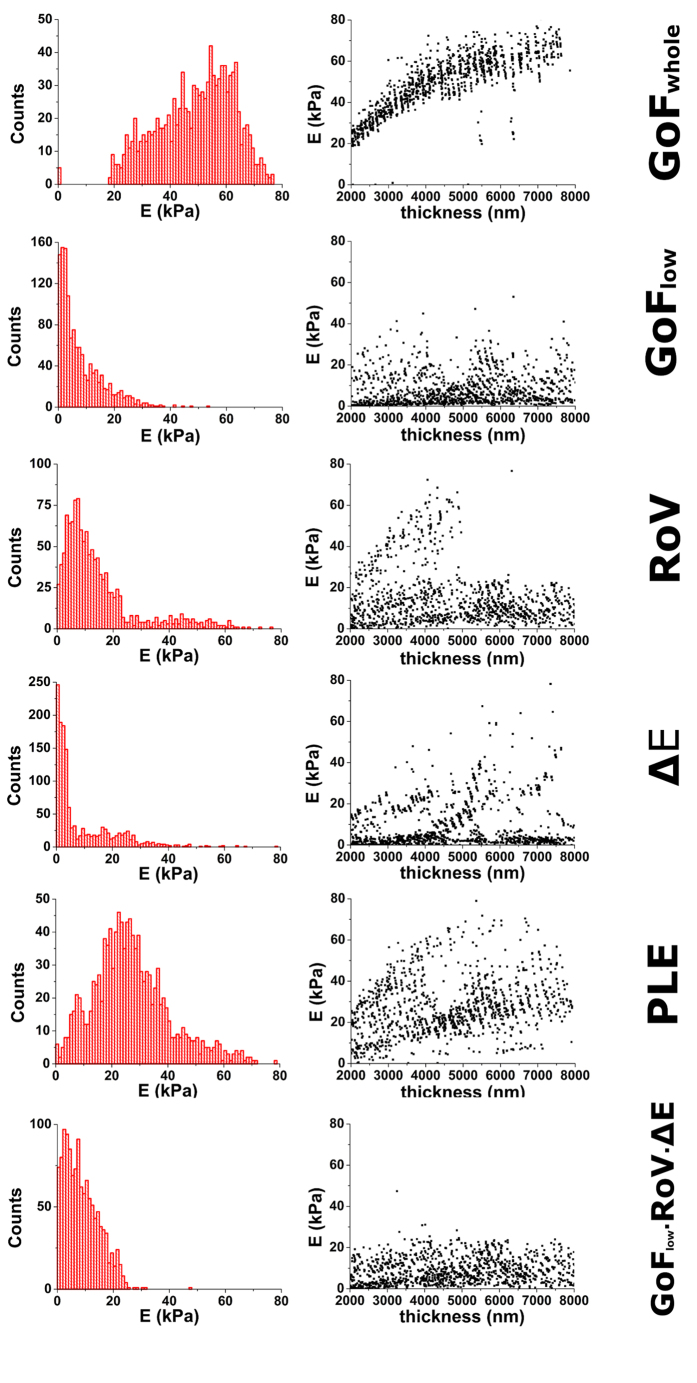
Performance of each strategy. (*Left column*) Distribution of Young’s modulus values computed using each search strategy for a collection of >1000 force-indentation curves obtained on thin ~10 kPa gels. (*Right column*) Relationship between computed *E* values and point thickness for each analysed force-indentation curve.

To test the performance of each strategy to identify the CP, we have used indentation curves obtained on thin homogeneous gels of graded thickness. In particular, we have focused on gel regions ranging from 2–8 μm in thickness, and have analysed >1000 indentation curves obtained on them. To measure success rate, we have defined the range of acceptable *E* values to be (0.3 kPa–60 kPa) for the case of 10 kPa gels, (0.15 kPa–9 kPa) for 1 kPa gels and (0.02 kPa–1 kPa) for 0.1 kPa gels. Figure 7 shows how different strategies yield significantly different distributions of *E* when analysing the same dataset obtained on 10 kPa thin gels (Suppl. Table 1). Several features are obvious when observing the resulting distributions of *E* values. On the one hand, the location of the peak of the distribution changes by up to an order of magnitude, as well as the spread of the distribution. Similarly, the skewness of the distribution also changes notably depending on the strategy used. Secondly, another remarkable difference between strategies is the ability (or lack thereof) to fully correct artifacts associated with the presence of the stiff substrate under the soft thin sample. In particular, GoF_whole_ and, to a less extend PLE, result in an ‘overcorrection’ of the bottom effect, where thinner areas appear to be softer than thicker ones.

To quantify differences in the performance of each strategy, we have used the assessment metrics defined above (Table 1). GoF_whole_ and PLE have the poorest performances, as reflected by the wide spread of the measured stiffness values (assessed as σ^2^(*E*)) and the residual dependence between point thickness and stiffness (assessed as σ(*E,h*)). As a result, their *M* values were the largest ones, indicating non-optimal performance of said strategies. On the contrary, GoF_low_ and ∆E yielded the best performance, resulting in very similar distributions of *E*. In both cases, peak values of *E* were markedly smaller than in the worst-performing strategies, and no dependence between point thickness and stiffness was observed. Nevertheless, it should be noted that both strategies (GoF_low_ and ∆E) yielded skewed *E* distributions.

We have also tested the possibility of combining more than one strategy, taking advantage of the fact that all of them are based on sequential searches. Table 2 shows the results for the best-performing combinations of 2, 3 or 4 strategies. Interestingly, the combination of GoF_low_ ∙ ∆E ∙ RoV yields the overall best performance on our data. For this combination, the distribution of *E* values has reduced spread and skewness, with no noticeable dependence between point thickness and stiffness (Fig. 7).

### Further verification on gels of different stiffness and thick samples

After identifying the best performing strategy (GoF_low_ ∙ RoV ∙ ∆E) on thin gels of 10 kPa stiffness, we have further tested whether it also provided improved results on thin gels of other stiffness (~0.1 kPa and ~1 kPa). Similar to the results above, GoF_whole_ has poor performance, displaying again a wide spread of reported stiffness values and a strong residual dependence between point thickness and stiffness (Suppl Tables 2 and 3). These issues are not observed when (GoF_low_ ∙ RoV ∙ ∆E) is used, and the overall performance, assessed using the parameter *M* is far superior to that obtained for GoF_whole_ (Suppl Tables 2 and 3). Also, for all gels tested, the bulk elastic modulus was similar to the average Young’s modulus measured by AFM when GoF_low_ ∙ RoV ∙ ∆E strategy was used to analyse hundreds of force-indentations curves. Conversely, using GoF_whole_ as strategy to identify the CP on the same force-indentation curves resulted in average Young’s modulus values consistently larger than the bulk elastic modulus (Table 3).

Finally, we have also assessed the performance of GoF_low_ ∙ RoV ∙ ∆E on indentation curves obtained on thick regions of the ~10 kPa stiffness hydrogel. In this case, we have used Sneddon’s model for an indenting cone to analyse the indentation curves. Interestingly, we see no marked difference between both strategies, which give rise to very similar *E* distributions. In addition, there is no overestimation of *E* when using GoF_whole_, and all the metrics to assess performance yield very similar values (Table 3 and Suppl Table 4).

### Application to adherent cells

**Figure 8:**
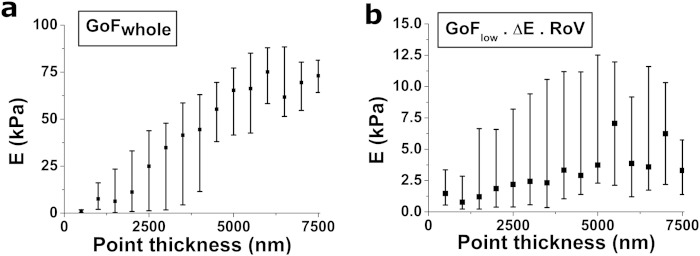
Application to force-indentation data on adherent cells. Force-indentation curves obtained on adherent cells (*n* = 24 cells, 300 indentations per cell) were analysed using (**a**) the most widely-used strategy GoF_whole_ versus (**b**) our optimal strategy GoF_low_ · RoV · ΔE. Young’s modulus values computed for each force-indentation curve are pooled together according to cell thickness at the probed location. Black squares indicate the median of the pooled data, while whiskers indicate 2.5% and 97.5% quantiles.

**Figure 9:**
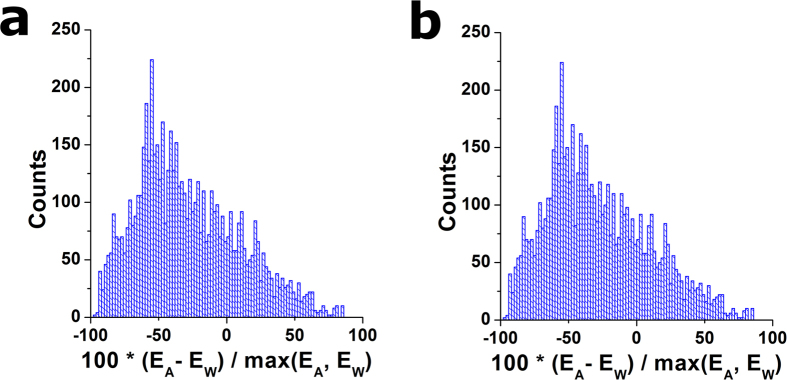
Features of viscoelasticity and depth-dependent stiffness behaviour in adherent cells. Force-indentation curves obtained on adherent cells were analysed using our optimal strategy GoF_low_ · RoV · ΔE to identify the CP, and further analysis was carried out as follows. For viscoelasticity (**a**) Young’s modulus values were computed for the approach and withdraw curves. For depth-dependent stiffness (**b**), Young’s modulus values where computed for low indentations (data interval 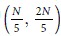) and high indentations (data interval 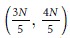), where *N* indicates the total number of data points for the contact part of the force-indentation curve. Results for 300 force-indentation curves per cell, carried out on *n* = 24 cells have been pooled together to generate the histograms.

Adherent cells are heterogeneous in gross morphology as well as in the composition and organization of their cytoskeleton. As a result, they display heterogeneous mechanical properties^7^,^18^,^19^. At the single cell level, the local mechanical properties are also heterogeneous^2^,^3^. Our results on soft hydrogels suggest that using GoF_whole_ to analyse data obtained on adherent cells is not optimal. In particular, we predict that GoF_whole_ will give rise to artifactually large intra-cell variability, stemming from the inherent variability in thickness along the cell’s surface^20^. Accordingly, we have compared the performance of the most standard strategy used in cell mechanics studies (GoF_whole_) with the optimal one (GoF_low_ ∙ ∆E ∙ RoV) (Table 4) by analysing force-indentation curves obtained on adherent cells (*n* = 24 cells, 300 indentations per cell). Measured cells displayed considerable variability in morphological parameters such as cell spread area (A = 1990 ± 970 μm^2^), and accordingly, we expected large variability in cell stiffness, both at the intra-cell and inter-cell levels. Nevertheless, intra-cell variability is markedly larger when GoF_whole_ is used. In particular, we observe again one of the pitfalls displayed by this strategy on thin hydrogels, that is, a strong dependence between the cell thickness at each probed location and the measured Young’s modulus (Fig. 8). Furthermore, and as previously reported^4^, GoF_whole_ also yields larger *E* values, and inter-cell variability is also increased. These data analysis artifacts were not observed when the same data set of force-indentation curves was processed using GoF_low_ ∙ ∆E ∙ RoV. Finally, our approach can also be extended to account for additional mechanical behaviours displayed by adherent cells, ie. viscoelasticity and depth-dependent stiffness. On the one hand, we have used our best-preforming strategy to analyse and compare the approach and withdraw curves in our measurements on adherent cells. As expected^21^, we observe that Young’s modulus values obtained for the withdraw curves are ~2 fold larger than for their approach curve counterparts (Fig. 9a). In addition, we have assessed depth-dependent mechanical properties of adherent cells utilizing a method previously described by us^22^. In this case, we can also reproduce the behaviour observed by others, in which shallow indentations result in larger observed Young’s modulus values than deeper indentations^23^ (Fig. 9b). Together, these results indicate that the optimal strategy we have previously identified using soft thin hydrogels yields indeed a superior performance when analysing indentation curves obtained in adherent cells.

## Discussion

When using GoF_low_ ∙ ∆E ∙ RoV as a CP search strategy, we obtain good agreement on gel stiffness values for measurements carried out in thin and thick samples using AFM. Furthermore, those values are also in agreement with bulk gel stiffness obtained using a materials test system. Said agreement reinforces the suitability of Sneddon’s and BECC models to analyse force-indentation data obtained with AFM on thin samples. Moreover, this approach can also be extended to the analysis of force indentation curves obtained on adherent cells, keeping in mind as inherent limitation that Hertzian models consider adherent cells as homogeneous and isotropic, linear elastic materials. While our results show that identifying correctly the CP in δ–F curves is crucial to obtain accurate estimates of Young’s modulus, especially in thin samples, other less significant sources of uncertainty still remain unchallenged in our study. In particular, neither Sneddon’s nor BECC account for the fact that standard AFM tips display pyramidal rather than conical shapes. Furthermore, some of the most used commercial AFM tips feature asymmetric pyramidal tips, further confounding the choice of a single opening angle to model the indentor. Nevertheless, strategies based one the Betti-Rayleigh reciprocal theorem^23^ may allow in the future to better adapt current models to the actual shape of AFM tips.

Force-indentation curves analysed in this study were performed on polyacrylamide gels of graded thickness, mimicking the height profile of adherent cells. It should be noted that the fabrication of such samples required some modifications to established protocols to prepare flat mm-thick polyacrylamide gels, in particular, avoiding the use a coverslip to sandwich the polyacrylamide drop between two glass surfaces. As a result, when polymerization was taking place our samples were more exposed to atmospheric oxygen than in standard protocols. Accordingly, while in our samples polymerization was not fully prevented by the increased presence of oxygen^24^, we routinely found that gel obtained using this protocol displayed stiffnesses lower than those reported by us and others for similar acrylamide and BIS conditions^25^,^26^.

Results obtained in thick gel areas (~mm thickness) display a distribution of *E* values that has very low spread and no noticeable skewness. As such, they illustrate the highest accuracy we can obtain with our experimental and data analysis approaches. Conversely, very thin samples such as adherent cells require more complicated data analysis models. In these experimental situations, not even our best performing strategy can achieve accuracy levels in the range of those obtained in thick gels. Of note, results obtained for our optimal strategy still display a log-normal distribution in *E* values, as opposed to the normal distribution expected for polyacrylamide gels. This effect is better explained by assessing how small changes in the identification of the CP are translated into over or under-estimation of *E.* In particular, using one of the example force indentation curves presented in Fig. 6, a 200 nm displacement of the CP towards the right of the δ–F curve changes the computed stiffness from 5.4 kPa to 7.8 kPa. Conversely, a 200 nm displacement of the CP towards the left lowers the computed stiffness to 3.5 kPa. In brief, and as already mentioned in section ‘Changes in estimated Young’s modulus (ΔE)’ above, *E*(*i*) follows a non-linear behaviour. Even in our best performing strategy, when using BECC in thin samples, the remaining small symmetric inaccuracies in the determination of CP will give rise to an asymmetric dispersion of *E* values which will resemble a log-normal distribution.

Computer processing time is a crucial issue when batch-processing the hundreds of force-indentation data generated by current AFM systems. All the CP identification strategies presented in this study are computationally expensive, because they are based on sequential searches that compute a test parameter for all candidate CPs along the δ–F curve (Table 5). Of note, RoV strategy is 100-fold faster than all other strategies tested, because its key numerical operation is computing the variance of a collection of points. Conversely, all other strategies require non-linear least-squares fitting of the data, which makes them more time intensitve. Of note, GoF_low_ ∙ ∆E ∙ RoV still requires only one single fitting step for each candidate CP, and therefore its use does not significantly increase the computation time required when compared to single strategies. To reduce computational cost, some sequential searches to identify the CP relay on the “golden section search”, a numerical technique first developed by Kiefer^27^. This method to identify numerically the maximum or minimum of a function greatly reduces the number of trial points assessed, thus significantly decreasing computation time. Nevertheless, the golden section search method can only be used in unimodal functions with a single extremum. As illustrated in Figs 1, 2, 3, 4, 5, this is not necessarily fulfilled in all our strategies, especially the ones based on combinations of them. Therefore, one drawback of using our optimal strategy (GoF_low_ ∙ ∆E ∙ RoV) is that all trial points within a test range have to be tested sequentially, thus increasing the computation time required to identify the CP. Strikingly, Fig. 1 shows that GoF_low_ does not yield an unimodal function when large enough search ranges are used. Such observation suggests the use of the golden section search technique^4^ may give rise to erroneous results, if a local maxima is incorrectly identified as the CP. Therefore, unless it can be guaranteed that *r*^*2*^ or the mean-squared error are unimodal functions as the trial point is marched along the f-d curve, a pre-search (or user input) may be required to limit the search range on the vicinity of the true CP.

Previous studies have proposed strategies to identify the CP of indentation curves. Nevertheless most of them have been limited to verifying that there were no major discrepancies between the location of the CP identified by novel and previous strategies^13^. As shown in Fig. 3a, a small change in the position of the CP may lead to a large change in reported *E* values, due to the non-linear relationship between CP and *E.* Therefore, we have directly measured differences in the distributions of *E*, rather than in the distributions of CP, to truly assess and compare the performance of different strategies. It should be noted that, for some of the metrics used, we have taken advantage of *a priori* knowledge of the expected distribution of *E.* In particular, the use of homogeneous hydrogels of known stiffness has allowed us to define a range of acceptable *E* values to compute a metric of success rate, as well as to establish *E* distribution skewness as another useful metric to assess strategies. Therefore, whenever possible, we recommend pre-testing the performance of strategies to identify the CP on homogeneous test samples with stiffness and topography similar to those exhibited by the samples of interest.

## Conclusion

We have presented several search strategies to identify the contact point of force-indentation curves obtained using Atomic Force Microscopy. To test them, we have used indentation curves obtained on elastic samples of constant stiffness, with topographies similar to those displayed by adherent cells. The large number of force-indentation curves analysed, and the mechanical homogeneity of our test samples has allowed us to use metrics based on the distribution of Young’s modulus values, to truly quantify the performance of each strategy. Taking advantage of the fact that all the proposed strategies are based on sequential search algorithms, we show that a combination of them yields the most accurate and unbiased results. It should be noted that the golden section search technique cannot be employed when using the optimal strategy proposed here, therefore making it a time-intensive procedure. While this study has focused on finding an optimal search strategy to analyse force-indentation curves on thin samples using the BECC model, said strategy is also accurate when analysing curves obtained on thick samples using Sneddon’s model.

## Additional Information

**How to cite this article**: Gavara, N. Combined strategies for optimal detection of the contact point in AFM force-indentation curves obtained on thin samples and adherent cells. *Sci. Rep.* **6**, 21267; doi: 10.1038/srep21267 (2016).

## Supplementary Material

Supplementary Information

## Figures and Tables

**Table 1:** Performance of the proposed strategies, including also GoF_whole_ as the most widely-used strategy in previous cell mechanics studies.

	*<E>* (kPa)	σ^2^(*E*) (kPa^2^)	SR	σ (*E,h*) (kPa·nm)	*s*(*E*)	M (kPa^3^·nm)
GoF_whole_	48.8	145	0.87	13300	−0.51	1.13 ∙ 10^6^
**GoF**_**low**_	**5.53**	**61**	**0.95**	**727**	**1.54**	**7.12 ∙ 10**^**4**^
RoV	10.41	170	0.99	−4780	1.78	1.47 ∙ 10^6^
∆E	3.96	118	0.89	−429	1.51	8.63 ∙ 10^4^
PLE	25.4	158	0.98	4960	0.52	4.14 ∙ 10^5^

A collection of >1000 force-indentation curves obtained on a thin ~10 kPa gel were analysed and the resulting distribution of *E* values were quantified using the assessment metrics defined in section 4. Highlighted in bold is the strategy that yielded the best results according to our assessment metrics.

**Table 2:** Performance of combined strategies.

	*<E>* (kPa)	σ^2^(*E*) (kPa^2^)	SR	σ (*E,h*) (kPa·nm)	*s*(*E*)	M (kPa^3^·nm)
GoF_low_ ∙ RoV	7.95	38	0.97	1020	0.98	3.95 ∙ 10^4^
**GoF**_**low**_ **∙ RoV∙∆E**	**7.90**	**46**	**0.99**	**825**	**0.83**	**3.54 ∙ 10**^**4**^
GoF_low_ ∙ RoV ∙ ∆E ∙ PLE	12.2	165	0.95	2490	2.05	8.89 ∙ 10^5^

Shown are only the strategies that performed best out of the possible combinations of 2, 3 or 4 of them. The dataset analysed was the same as in Table 1. Highlighted in bold is the strategy that yielded the overall best results according to our assessment metrics.

**Table 3:** Further application to other gel stiffnesses.

*<E>* (kPa)	GoF_whole_	GoF_low_ ∙ RoV ∙ ∆E	Bulk
~10 kPa	48 ± 12	7.9 ± 6.8	11.1 ± 1.4
~1 kPa	6.4 ± 3.1	0.71 ± 1.3	1.7 ± 1.3
~0.1 kPa	0.34 ± 0.22	0.10 ± 0.08	0.26 ± 0.09

Average Young’s modulus computed using the most widely-used strategy (GoF_whole_) and our optimal strategy (GoF_low_ · RoV · ΔE) on a collection of >1000 force-indentation curves obtained on thin gels of different stiffness. For comparison, bulk mechanical properties for the same gel preparations are also shown (1 gel each, 15 indentations per gel). Data presented as mean ± SD.

**Table 4:** Comparison between the performance of the most widely-used strategy (GoF_whole_) versus our optimal strategy (GoF_low_ · RoV · ΔE) when analysing force-indentation curves obtained on adherent cells (*n* = 24 cells, 300 indentations per cell).

	*<E>* (kPa)	Inter-cell variability	Intra-cell variability
GoF_whole_	15.4	78%	119 ± 82%
GoF_low_ ∙ RoV ∙ ∆E	1.97	54%	77 ± 28%

**Table 5:** Computation time for different tested strategies.

	Time (sec)
GoF_whole_	3.5 ± 0.4
GoF_low_	3.6 ± 0.6
RoV	0.016 ± 0.007
∆E	3.8 ± 0.8
PLE	3.1 ± 0.4
GoF_low_ ∙ RoV ∙ ∆E	3.9 ± 0.7

Computation times presented here were obtained when our custom-built Matlab code was run on a personal computer equipped with an Intel® Core™ i7-3770 processor running at 3.4 GHz. Values correspond to the average time required to find the CP in one force-displacement curve, disregarding additional processes such as file reading/writing or data plotting. To produce the averages, a collection of >1000 force-indentation curves obtained on a thin ~10 kPa gel were analysed. Data is presented as mean ± SD.
