# Supplementary material for: Combined strategies for optimal detection of the contact point in AFM force-indentation curves obtained on thin samples and adherent cells
Source: Sci Rep. 2016 Feb 19;6:21267. doi: 10.1038/srep21267 (PMC4759531; doi:10.1038/srep21267)
Supplement: Supplementary Information [file srep21267-s1.docx]

**Supplementary Information**

**Combined strategies for optimal detection of the contact point in AFM force-indentation curves obtained on thin samples and adherent cells.**

Núria Gavara^*^

School of Engineering and Materials Science, Queen Mary University of London, Mile End Road, E1 3NS, London, UK.

^*^e-mail: [n.gavara@qmul.ac.uk](mailto:n.gavara@qmul.ac.uk)

**Supplementary tables**

**Suppl. Table 1.** Statistical differences between *E* distributions yielded by different strategies. A collection of >1000 force-indentation curves obtained on a thin 10kPa gel were used, and the resulting distributions obtained for each strategy were tested. To do so, we used bootstrapping to generate distributions of 1000 mean, variance and skewness values for each strategy. The resulting distributions were then compared using two-way ANOVA analysis (mean, variance or skewness, as 1^st^ factor, strategy used as 2^nd^ factor) followed by a Bonferroni test for multiple comparisons. Boxes indicate the p-value for comparisons accounting only for differences in the 2^nd^ factor (strategy used). Grey shaded boxes indicate identity.

|  | GoF_whole_ | GoF_low_ | RoV | ∆E | PLE | GoF_low_ ∙ RoV ∙ ∆E |
| --- | --- | --- | --- | --- | --- | --- |
| GoF_whole_ |  | 0 | 10^-23^ | 0 | 0 | ^0^ |
| GoF_low_ |  |  | 0 | 10^-96^ | 0 | 10^-36^ |
| RoV |  |  |  | 0 | 10^-189^ | 0 |
| ∆E |  |  |  |  | 10^-173^ | 10^-15^ |
| PLE |  |  |  |  |  | 10^-284^ |

**Suppl. Table 2.** Comparison between the performance of the proposed strategies on a collection of >1000 force-indentation curves obtained on a thin 1kPa gel.

|  | *<E>* (kPa) | σ^2^(*E*)  (kPa^2^) | SR | σ(*E,h*)  (kPa·nm) | *s*(*E*) | M  (kPa^3^·nm) |
| --- | --- | --- | --- | --- | --- | --- |
| GoF_whole_ | 6.35 | 9.88 | 0.94 | 1410 | 0.70 | 1.04 ∙ 10^4^ |
| GoF_low_ | 0.46 | 0.64 | 0.81 | 52 | 3.86 | 1.58 ∙ 10^2^ |
| RoV | 1.76 | 13.11 | 0.72 | 423 | 1.39 | 1.07 ∙ 10^4^ |
| ∆E | 1.54 | 79.2 | 0.87 | 187 | 17.76 | 3.02 ∙ 10^5^ |
| PLE | 0.66 | 1.41 | 0.78 | 135 | 3.54 | 8.63 ∙ 10^2^ |
| GoF_low_ ∙ RoV | 0.73 | 1.34 | 0.87 | 86 | 2.39 | 3.16 ∙ 10^2^ |
| GoF_low_ ∙ RoV∙∆E | 0.71 | 1.28 | 0.96 | 74 | 1.65 | 1.50 ∙ 10^2^ |
| GoF_low_ ∙ RoV ∙ ∆E ∙ PLE | 1.02 | 1.77 | 0.95 | 66 | 1.4261 | 1.75 ∙ 10^2^ |

**Suppl. Table 3.** Comparison between the performance of the proposed strategies on a collection of >1000 force-indentation curves obtained on a thin 0.1kPa gel.

|  | *<E>* (kPa) | σ^2^(*E*)  (kPa^2^) | SR | σ(*E,h*)  (kPa·nm) | *s*(*E*) | M  (kPa^3^·nm) |
| --- | --- | --- | --- | --- | --- | --- |
| GoF_whole_ | 0.34 | 0.049 | 0.90 | 121 | 0.56 | 3.68 ∙ 10^0^ |
| GoF_low_ | 0.06 | 0.007 | 0.89 | -116 | 2.03 | 1.85 ∙ 10^0^ |
| RoV | 0.16 | 0.018 | 0.92 | -179 | 0.77 | 2.70 ∙ 10^0^ |
| ∆E | 0.13 | 0.009 | 0.90 | -162 | 1.33 | 2.15 ∙ 10^0^ |
| PLE | 0.12 | 0.019 | 0.88 | -210 | 1.92 | 8.71 ∙ 10^0^ |
| GoF_low_ ∙ RoV | 0.09 | 0.008 | 0.97 | -81 | 1.77 | 1.33 ∙ 10^0^ |
| GoF_low_ ∙ RoV ∙ ∆E | 0.10 | 0.006 | 0.96 | -44 | 1.71 | 4.56 ∙ 10^-1^ |
| GoF_low_ ∙ RoV ∙ ∆E ∙ PLE | 0.09 | 0.008 | 0.99 | -84 | 1.02 | 6.92 ∙ 10^-1^ |

**Suppl Table 4.** Comparison between the performance of the most widely-used strategy (GoF_whole_) versus our optimal strategy (GoF_low_ · RoV · ΔE ) on a collection of >1000 force-indentation curves obtained on a thick region (thickness ~ 2mm) of a 10kPa gel.

|  | *<E>* (kPa) | σ^2^(*E*)  (kPa^2^) | SR | σ(*E,h*)  (kPa·nm) | *s*(*E*) | M  (kPa^3^·nm) |
| --- | --- | --- | --- | --- | --- | --- |
| GoF_whole_ | 10.5 | 0.61 | 0.95 | 45 | -0.30 | 8.56 ∙ 10^0^ |
| **GoF_low_ ∙ RoV ∙ ∆E** | **10.2** | **0.76** | **0.95** | **27** | **-0.33** | **7.16 ∙ 10^0^** |
